# Reaction of Lectin-Specific Antibody with Human Tissue: Possible Contributions to Autoimmunity

**DOI:** 10.1155/2020/1438957

**Published:** 2020-02-11

**Authors:** Aristo Vojdani, Daniel Afar, Elroy Vojdani

**Affiliations:** ^1^Immunosciences Lab, 822 S. Robertson Blvd., Ste. 312, Los Angeles, CA 90035, USA; ^2^University of California, Los Angeles, CA 90095, USA; ^3^Regenera Medical, 11860 Wilshire Blvd., Ste. 301, Los Angeles, CA 90025, USA

## Abstract

The aim of this study was to examine the direct reaction of specific lectin/agglutinin antibodies to different tissue antigens to confirm the theory that reactivity between them may contribute to autoimmunities. Lectins are carbohydrate-binding proteins found in nearly all fruits and vegetables. Undigested lectins can penetrate the gut barriers, provoking an immune response that results in the production of antibodies against them. Using an enzyme-linked immunosorbent assay, we reacted lectin-specific antibodies with 62 different tissue antigens. Wheat germ agglutinin-specific antibody was the most reactive with the tissue antigens (37 tissues out of 62), followed by red kidney bean phytohemagglutinin-specific antibody (20), soybean agglutinin-specific antibody (20), and peanut agglutinin-specific antibody (15). This reaction between anti-lectin antibodies and many human tissue antigens may be due to possible molecular mimicry and cross-reactivity. After our results confirmed that anti-lectin antibodies bind with human tissues, we wanted to determine the prevalence of these antibodies in the blood of 500 nominally healthy donors. The percentage elevation of antibodies against different lectins ranged from 12 to 16% (Immunoglobulin G), 9.7-14.7% (Immunoglobulin A), 12-18% (Immunoglobulin M), and 7.8-14.6% (Immunoglobulin E). Serial dilutions and inhibition study confirmed that these reactions were specific. Finally, we tested the lectin-specific antibody level in sera both negative and positive for RF and ANA and found that IgM anti-lectin antibody levels were highly correlated with RF but not with ANA level. The reaction of anti-lectin antibodies with human tissue components and their detection in RF-positive samples may describe mechanisms by which the production of antibodies against undigested lectins may contribute to the pathogenesis of some autoimmune diseases.

## 1. Introduction

Autoimmune disease occurs when the human body loses its tolerance mechanism and its ability to discriminate between self and nonself molecules. This loss of oral, central, and peripheral tolerance may ultimately result in the destruction of self-tissue by both autoantibodies and autoreactive T-lymphocytes [1]. Most autoimmune diseases develop due to genetics and environmental factors such as food antigens, haptenic chemicals that form neo-antigens, and different pathogens [2]. The exact mechanisms have yet to be clearly defined. However, in the past 30 years, the process of molecular mimicry has gained increasing recognition by investigators [3–12]. For example, studies have found that autoantigens known to be involved in autoimmune diseases shared sequence homology with food items such as wheat and milk, as well as with aquaporin-containing foods such as soy, corn, tomato, and spinach [7, 12].

In addition to molecular mimicry, food components such as lectins and agglutinins may contribute to autoimmune diseases by directly binding to human tissue and components of the gut microbiome [13–17]. Lectins/agglutinins are ubiquitous carbohydrate-binding proteins that are found in animals, plants, and microorganisms. They perform recognition functions on many biological levels. In animals, they regulate cell adhesion, glycoprotein synthesis, and blood protein levels, and play important roles in the innate immune system, mediating the first line of defense against invading microorganisms. They play a role in nonself recognition and are probably also involved in inflammatory and autoreactive processes [18]. Unfortunately, undigested food lectins that manage to penetrate the barriers can have devastating consequences for the host. Both gut bacteria and epithelial cells carry receptors for different lectins. This enables lectins to bind to gut bacteria, gut epithelial cells, or both. The binding of lectins to epithelial cells may cause inflammation, damage to the tight junctions, and leaky gut, which has been proposed as the gateway to autoimmunity [19–21]. Dietary lectins may also induce the release of endotoxins such as lipopolysaccharides (LPS), which increase gut permeability, thus allowing the entry of lectins, food antigens, and bacterial toxins into the circulatory system. Furthermore, a large number of human tissues express molecules that act as lectin receptors. Lectins can home into these receptors and bind to this diverse group of multiple target tissues, which includes connective tissue, the liver, pancreas, thyroid, cardiac muscle, prostrate, breast, and brain [22]. This binding of lectins to different tissue antigens can activate cellular and humoral responses against both lectins and the specific tissues to which the lectins manage to bind.

For instance, if lectins bind to islet cells of the pancreas, an autoimmune response can be launched against the islet cells, contributing to type 1 diabetes. Lectins can also bind to the major joint components glycosaminoglycans and proteoglycans, possibly leading to autoimmune rheumatic disorders. This is because about 3 hours after consumption, the digestive-resistant dietary lectins in intact form are able to cross the intestinal barrier and enter the circulation, through which they can reach the joints and interact with the synovial tissue [23]. In a rabbit model of arthritis, the injection of dietary lectins into the knee joint induced the development of severe arthritis characterized by amplification of inflammatory responses [24]. Furthermore, in another study [25], researchers found that IgG purified from the blood of rheumatoid arthritis (RA) patients had the capacity to bind to several lectins up to 50-fold greater than the binding capability of IgG from controls. It was hypothesized that this greater binding capacity was due to a specific galactosylation deficiency in the oligosaccharide chain of the IgG from the RA patients resulting in IgG aggregation and the forming of rheumatoid factor or IgM anti-IgG. Lectins can also bind to the glomerular basement membrane, with the resulting autoimmune response causing glomerulonephritis [21].

Additionally, lectins may bind as well to human endometrium, spermatozoa, and ova. An autoimmune reaction against these targets could cause infertility in men or women [22, 26–29].

The following are major lectins known to be involved in the pathogenesis of some autoimmune diseases: wheat germ agglutinin (WGA), red kidney bean phytohemagglutinin (PHA), jack bean agglutinin (ConA), peanut agglutinin (PNA), and soybean agglutinin (SBA). Although lectin has been found in wheat, rye, barley, oats, corn, and rice, WGA is the best studied of the cereal grain lectins [30]. In one study, the administration of WGA to experimental animals caused hyperplastic and hypertrophic growth of the small intestine and pancreas and atrophy of the thymus [31].

Many cell surface-exposed glycoconjugates have at their terminal positions a wide family of 9-carbon sugars called sialic acids, which are used for self-recognition in the vertebrate immune system. Unfortunately, they can also be used as a binding target for pathogenic, extrinsic receptors and molecular toxins [32]. WGA is able to bind to N-acetylneuraminic acid (Neu5Ac), the sialic acid predominantly found in human cells, and thus, it is able to adhere to cell surfaces such as the epithelial layer of the gut [32, 33]. The surface of many prokaryotic and eukaryotic cells is covered with a dense coating of glycoconjugates or glycocalyx. The binding of WGA to Neu5Ac at the glycocalyces of human cells and pathogens expressing Neu5Ac allows for cell entry and could induce a proinflammatory immune response, especially at the gastrointestinal surfaces [16, 34–36].

Furthermore, WGA is capable of activating NF-*κ*B proteins which, when upregulated, are involved in practically every acute and chronic inflammatory disorder, including neurodegenerative, inflammatory bowel, and autoimmune diseases [37]. Similarly, high levels of soybean agglutinin (SBA) have been shown to have toxic effects on mammalian organs and metabolism, including induced growth of the small intestine and pancreas, depletion of lipid in carcass, atrophy of spleen and kidney, and reduction in circulating insulin [38]. PHA is a lectin isolated from red kidney beans. This hemagglutinin, along with ConA from jack beans, has the capacity to agglutinate mammalian erythrocytes; additionally, by binding to the membranes of T cells, these lectins can stimulate metabolic activity, resulting in cell proliferation [39]. PNA is a nonglycosylated protein that has high affinity for the T-antigen structure, specifically the D-galactose residues, and can be used to distinguish between lymphocyte subsets [40].

The binding of different lectins to a variety of cell surfaces is made possible by the lectins' different carbohydrate specificities. For example, WGA, SBA, rice, and barley are sugar-specific to N-acetyl-D-glucosamine; mushroom, zucchini, and string beans to N-acetyl-D-galactosamine; PNA to D-galactose; ConA to methyl-mannoside or methyl glucoside; lentil sprouts to D-methyl-mannoside or D-glucose; and PHA to N-acetylglucosamine [21]. The interaction between undigested lectins and different carbohydrate groups on different cell types can result in the activation of the immune system to react to the lectins as well as to the tissues to which the lectins have managed to bind.

The mechanisms by which lectins induce autoimmunity are currently described based largely on the direct interaction of lectins with gut microbiota, the release of bacterial toxins, the increased intestinal permeability, and the passage of dietary lectins and other food antigens into the periphery. The aims of this particular study was to examine the direct reaction of lectin-specific antibodies with a variety of human tissue antigens; to find the prevalence of IgG, IgA, IgM, and IgE antibodies against six different lectins in the sera of healthy donors; and, finally, to measure anti-lectin antibodies in sera that were either positive or negative for rheumatoid factor (RF) or anti-nuclear antibody (ANA). The reaction of anti-lectin antibodies with human tissue components and their detection in samples positive for RF and/or ANA may describe additional mechanisms by which the production of antibodies against undigested lectins contribute to the pathogenesis of autoimmune diseases.

## 2. Materials and Methods

### 2.1. Blood Samples

Sera from 500 healthy individuals who were qualified to donate blood (264 males of different ethnicities aged 18-65 years with a median age of 39.5 years, and 236 females of different ethnicities aged 18-65 with a median age of 38.2 years) were obtained from Innovative Research, Inc. (Southfield, MI, USA). The subjects were tested according to FDA guidelines for the detection of hepatitis B surface antigen, and antibodies to HIV, HIV-1 RNA, Hepatitis-C RNA, and syphilis. All samples yielded nonreactive or negative results for each test performed. Samples were screened for the level of RF and ANA; lectin-specific antibodies were measured in 48 RF-positive, 48 RF-negative, 48 ANA-positive, and 48 ANA-negative samples.

### 2.2. Antibodies to Lectins and Agglutinins

Wheat germ agglutinin (WGA), peanut agglutinin (PNA), soy bean agglutinin (SBA), phytohemagglutinin (PHA-E+L), lentil lectin, pea lectin, and affinity-purified polyclonal goat anti-lectin antibodies were purchased from Vector Laboratories (Burlingame, CA, USA).

### 2.3. Proteins and Peptides

Parietal cell, intrinsic factor, fibrinogen, laminin, thyroglobulin, neutrophil cytoplasmic antigen, and collagen type V were purchased from MP Biologicals (Solon, OH, USA).

Recombinant thyroid peroxide was purchased from Fitzgerald (Acton, MA, USA).

Cardiolipin, actin, myelin basic protein (MBP), tropomyosin, ganglioside GM1, insulin, hepatocytes, transglutaminase-2, transglutaminase-3, transglutaminase-6, enolase, amyloid-beta peptide, tau protein, somatotropin, human serum albumin (HSA), dipeptidylpeptidase IV (DPP-IV), *Saccharomyces cerevisiae*, alpha-myosin, calprotectin, tyrosinase, elastin, motilin, vasoactive intestinal peptide (VIP), myoglobin, fibrinogen, and lysosome were purchased from Sigma-Aldrich (St. Louis, MO, USA).

Glial fibrillary acidic protein (GFAP), brain-derived neurotrophic factor (BDNF), beta nerve growth factor (beta-NGF), S100B protein, calmodulin, platelet glycoprotein, *α*-synuclein, acetylcholine receptor, and elastase were purchased from Calbiochem (San Diego, CA, USA).

Different peptides of occludin, zonulin, claudin-5, E-cadherin, vinculin, aquaporin-4 (AQP4), presenilin, fibulin, protein disulfide isomerase (PDI), cerebellar, rabaptin-5 (rab5), enteric nerve neuronal nuclear antigen (enteric nerve NNA), glutamate receptor (glutamate-R), dopamine receptor (dopamine-R), glutamic acid decarboxylase 65 (GAD-65), 21-hydroxylase, ovary, islet cell antigen, arthritis-induced peptide, alpha+beta tubulin, synapsin, neurocrescin, and neurotrophin, all with a purity greater than 90%, were synthesized by Bio-Synthesis (Lewisville, TX, USA).

### 2.4. Reaction of Different Lectin/Agglutinin-Specific Antibodies with Tissue Antigens by ELISA

100 *μ*l of different tissue antigens and peptides at a concentration of 10 *μ*g/mL in 0.1 M of borate buffer pH 9.2 were added to quadruplicate wells of each microtiter plate and incubated for 8 hrs at 25°C, followed by incubation for 16 hrs at 4°C. The plates were washed, the unoccupied wells were coated with 200 *μ*L of 2% BSA and 1% dry milk, and then, the plates were incubated for 24 hrs at 4°C. The plates were washed again, and 100 *μ*L of affinity-purified goat polyclonal anti-WGA antibodies, anti-PNA antibodies, anti-SBA antibodies, or anti-PHA antibodies diluted 1 : 100 in 1% BSA in 0.1 M phosphate buffer saline (PBS) pH 7.4 were added to different sets of microtiter plates that were coated with tissue antigens. This procedure was followed by a repeated washing and the addition of 1 : 200 dilution of alkaline-phosphatase-labeled anti-goat polyclonal antibody to all wells including the controls; after which, plates were incubated again for 1 hr at 25°C. After a final washing and the addition of substrate, plates were incubated again for 30 mins at 25°C, after which, 60 *μ*L of stop solution was added to all wells, followed by measurement of the ODs.

### 2.5. Demonstration of Specificity of Anti-Lectin/Agglutinin-Specific Antibody Binding to Tissue Antigens

We chose three different tissue antigens with which the anti-lectin antibodies had moderate to strong reactions. To demonstrate the specificity of these antibody-antigen reactions, we performed both serial dilution and inhibition studies.

For serial dilutions, anti-WGA, anti-PNA, anti-SBA, or anti-PHA antibodies serially diluted from 1 : 100 to 1 : 12,800 were added to different strips of microtiter plates coated with thyroid peroxidase (TPO), *α*-myosin, or ganglioside. After incubation, washing and repeat of all other ELISA steps, optical densities were measured.

For inhibition study, different sets of four rows of microtiter plates were coated with WGA, PNA, SBA, or PHA. To the first row of all wells, 100 *μ*l of diluent was added. To the additional rows of the lectin-coated wells, 50 *μ*l of PBS containing 1.5-96 *μ*g of HSA, TPO, *α*-myosin, or asialoganglioside was added, respectively. 100 *μ*l of anti-WGA antibodies was then added to all wells of the first set of four strips. Similarly, to the additional sets of microwell strips, 100 *μ*l of PNA, SBA, or PHA-specific antibody was added. Plates were kept on the shaker for 5 minutes and were incubated for an additional hour at RT. After washing and addition of the secondary antibody, repeat of incubation, washing, and the addition of substrate, ODs were recorded, and indices were calculated.

### 2.6. Examination of Cross-Reactivity between Different Lectins/Agglutinins

We reacted anti-WGA-, anti-PNA-, anti-SBA-, and anti-PHA-specific antibodies with ELISA plates coated with 1 g of lectins from WGA, PNA, SBA, PHA, lentil, and pea. After completion of all ELISA steps, the optical densities were recorded, and the indices calculated.

### 2.7. Measurement of Antibodies against Lectins/Agglutinins by Enzyme-Linked ImmunoSorbent Assay (ELISA)

One mg of different lectins/agglutinins was dissolved in 1 mL of 0.1 M PBS at pH 7.4. Next, these antigens were diluted 1 : 50 in the same buffer, and 100 *μ*L or 2 *μ*g of each antigen was added to the wells of a microtiter plate, which was then incubated overnight at 4°C. After washing, the unoccupied wells were saturated by adding 200 *μ*L of 2% bovine serum albumin (BSA), and the plates were incubated overnight again at 4°C. The plates were washed again, and 100 *μ*L of different sera diluted at 1 : 4 for IgE and 1 : 100 for IgG and IgA was added to duplicate wells of each microtiter plate and incubated for 1 hr at room temperature (RT). This procedure was followed by washing and the addition of alkaline-phosphatase-labeled anti-human IgE at a dilution of 1 : 200 to 1 set and anti-human IgG, IgM, or IgA at a dilution of 1 : 400 in serum diluent to a different set of plates, followed by incubation for 1 hr at RT. After another washing and the addition of a substrate to the wells, the color development was measured at 405 nm. Four sera from patients with a known allergy to different lectins and four sera from individuals with no known allergies to lectins were used as calibrators and positive and negative controls. Several wells were coated with unrelated proteins, such as human serum albumin (HSA), rabbit serum albumin (RSA), and BSA; these were used only for the determination of the ELISA background. The following formula was used for the calculation of antibody indices:

(1) $$\begin{array}{c} \text{Antibody index}=\frac{\text{OD of sample}–\text{OD of background}}{\text{OD of calibrator}–\text{OD of background}} \end{array}$$

### 2.8. Kits for Detection of RF and ANA

For the determination of RF by IU, kits were purchased from INOVA Diagnostics (San Diego, CA, USA). The ANA titer was measured by Colorzyme® slides purchased from Immuno Concepts (Sacramento, CA, USA). Using the same ELISA methodology, IgG and IgM were measured against the same six lectins in 48 samples with an RF level ranging from 0 to 5 IU/mL, and the results were compared with 48 samples ranging from 101 to 342 IU. IgG antibody against these same six lectins was also measured in 48 sera with the ANA titer of 20 or less, and the results were compared to those from 48 additional specimens with the ANA titer ranging from 1 : 80 to 1 : 1280.

### 2.9. Statistical Analysis

Statistical analysis using Microsoft Excel's *t* test function was performed. Comparison of the ODs of all wells used as controls to the ODs of the antibody reactivity of the four lectins against all tested tissue antigens was performed. A Bonferroni adjustment account for type 1 errors with multiple comparisons was set to adjust the alpha (*p* > 0.001). We calculated Pearson's correlation coefficient between ANA, rheumatoid factor IgG, and IgM isotype antibodies detected in the same specimens. We then performed a simple regression analysis between each of these combinations and calculated their *p* values. Finally, we carried out a two-way cluster analysis of Pearson's correlation coefficient between ANA and RF levels with different lectin antibodies. All statistical tests and the two-way cluster analysis were performed using statistical software “R.”

## 3. Results

### 3.1. Reaction of Affinity-Purified Lectin/Agglutinin Antibodies with Different Tissue Antigens

We measured the degree of immune reactivity of the affinity-purified polyclonal goat anti-WGA, anti-PNA, anti-SBA, and anti-PHA antibodies, and unimmunized goat serum with 62 different tissue antigens using ELISA methodologies. The ELISA indices for all these reactions were within 3 SD above the mean ODs of control wells (OD 0.43) containing all the reagents but not goat serum. To interpret the results, we used the following key: index of which was established based on the mean ± 3 SD of control wells that contained all reagents but not the first antibody: 0.43-0.80 = + (borderline); 0.81-1.3 = ++ (low); 1.31-2.0 = +++ (moderate); 2.1-3.0 = ++++ (strong); >3 = +++++ (very strong).

We first found that the control goat serum reacted only to somatotropin with a very low index of 0.48 and did not react with the other 61 tissue antigens. However, anti-WGA antibody reacted very strongly with TPO (index 3.4) and strongly with zonulin, calmodulin, ovary, insulin+islet cell, and neurocrescin in a descending order of strength of reaction with indices ranging from 2.7 to 2.2. With acetylcholine-R, presenilin, MBP, tau protein, ASCA+ANCA, and enolase, the reaction was moderate (index from 1.43 to 2.0); with 18 different tissue antigens, the indices were low (0.9-1.3); and with the remaining tissue antigens, the anti-WGA antibody reactivity ranged from negative to borderline (Table 1). Reaction of anti-PHA antibody with TPO, similar to anti-WGA antibody, was also very strong (index 3.6), strong with asialoganglioside (index 2.9) and hepatocytes (2.3), and moderate with the following in decreasing strength of reactivity: intrinsic factor, calprotectin, *α*-myosin, tyrosinase, neurofilaments, and calmodulin, with indices ranging from 2.0 to 1.34. With 14 different tissue antigens, the reactions were borderline to low with indices ranging from 0.53 to 1.30, and with another 39 different tissue antigens, the anti-PHA antibody reacted with indices < 0.43. In comparison, the anti-PNA antibody had a low reaction with dopamine-R (1.0) and glutamate-R (1.1), 17 other antigens had borderline or one plus reactions, and the other 43 antigens had indices that were below 0.43 (insignificant). The anti-SBA antibody reacted moderately with tyrosinase with an index of 1.6; with 20 different tissue antigens, the reactions were borderline to low with indices ranging from 0.44 to 0.9; and with another 41 different tissue antigens, the anti-SBA antibody reacted with indices < 0.43 (Table 1).

For WGA-specific antibodies, the *p* values are listed as *p* > 0.05 for 25 different tissue antigens and highly significant *p* values (*p* < 0.00001) for 37 antigens. For PNA-specific antibodies, the *p* values are listed as *p* > 0.05 for 43 different tissue antigens and highly significant *p* values (*p* < 0.00001) for 19 antigens. For SBA-specific antibodies, the *p* values are listed as *p* > 0.05 for 41 different tissue antigens and highly significant *p* values (*p* < 0.00001) for 21 antigens. For PHA-specific antibodies, the *p* values are listed as *p* > 0.05 for 39 different tissue antigens and highly significant *p* values (*p* < 0.00001) for 23 antigens.

### 3.2. Demonstration of Specificity of Anti-Lectin Antibody Binding to Tissue Antigens

**Figure 1:**
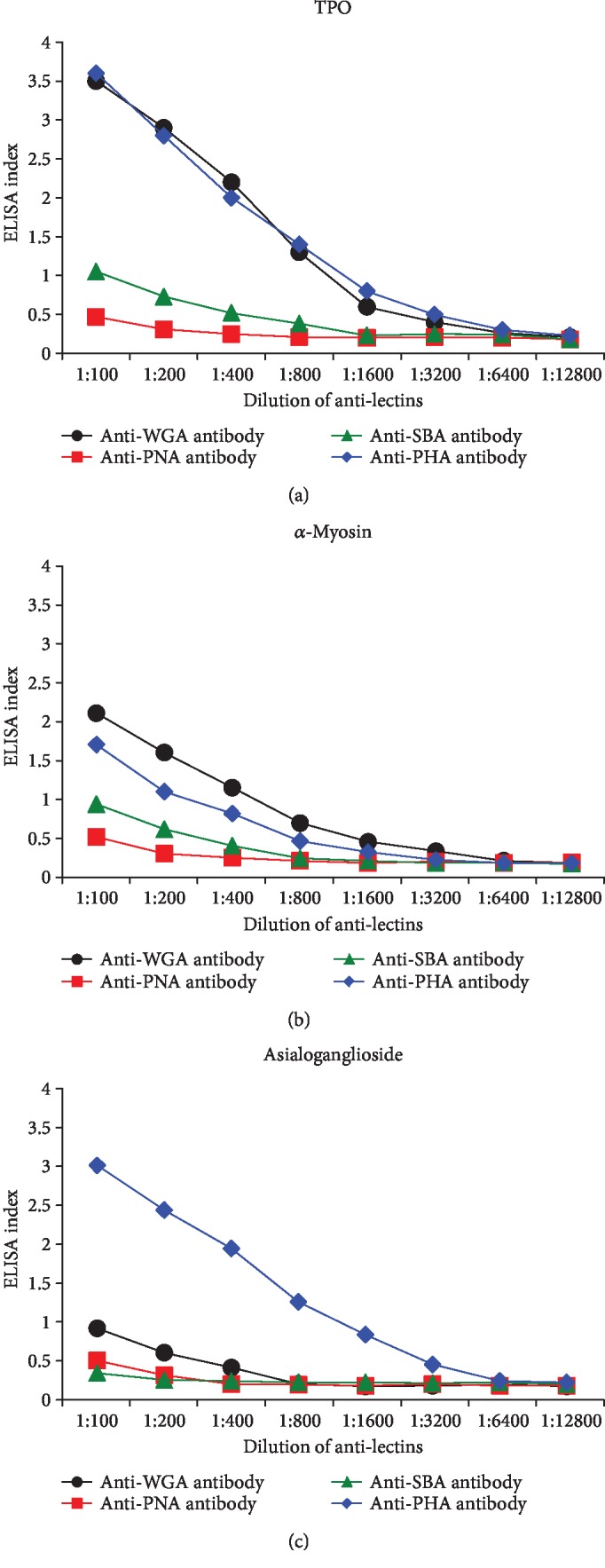
(a) Binding of serially diluted anti-lectin antibodies to TPO-coated microplate wells. (b) Binding of serially diluted anti-lectin antibodies to *α*-myosin-coated microplate wells. (c) Binding of serially diluted anti-lectin antibodies to asialoganglioside GM_1_-coated microplate wells.

To demonstrate the specificity of anti-lectin antibodies binding to different tissue antigens, serial dilution and inhibition studies were performed. The addition of WGA-, PNA-, SBA-, and PHA-specific antibodies diluted from 1 : 100 to 1 : 12800 to TPO, *α*-myosin, and ganglioside-coated plates followed by the other required ELISA steps resulted in a significant decline in antibody indices in proportion to the antibody dilution. This decline was significant with anti-WGA and anti-PHA antibody reaction with TPO, *α*-myosin, and ganglioside (Figures 1(a)–1(c)) up to a dilution of 1 : 3200. Antibodies that had a low reaction with tissue antigens showed a decline of only up to 1 : 400 dilution.

**Figure 2:**
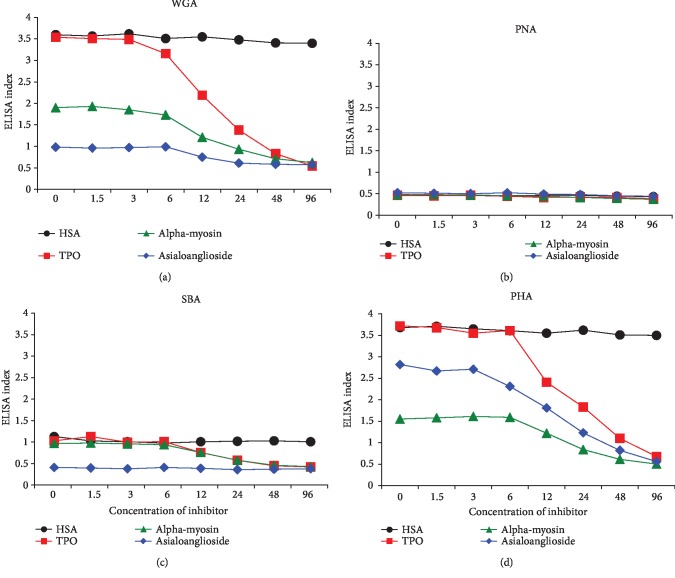
Inhibition of different anti-lectin antibodies with different lectins and agglutinins. (a) Reaction of anti-WGA antibody binding to WGA-coated plates in the presence of HSA, TPO, *α*-myosin, and asialoganglioside, respectively. (b) Reaction of anti-PNA antibodies binding to PNA-coated plates in the presence of HSA, TPO, *α*-myosin, and asialoganglioside, respectively. (c) Reaction of anti-SBA antibodies binding to SBA-coated plates in the presence of HSA, TPO, *α*-myosin, and asialoganglioside, respectively. (d) Reaction of anti-PHA antibodies binding to PHA-coated plates in the presence of HSA, TPO, *α*-myosin, and asialoganglioside, respectively.

For the inhibition study, the anti-lectin antibody reactions to TPO, *α*-myosin, and asialoganglioside were chosen based on the strength of their reactivity with either anti-WGA antibody or anti-PHA antibody or both. Different amounts of specific antigens (TPO, *α*-myosin, asialoganglioside, and HSA as control) in concentrations ranging from 0 to 96 *μ*g were added to the liquid phase of ELISA wells that were coated with lectins. Compared to HSA or control protein that did not cause any inhibition in these antibody-antigen reactions, the addition of higher concentrations of specific tissue antigens to the liquid phase, followed by the addition of lectin-specific antibodies, resulted in a significant decline in the binding of anti-lectin antibodies to lectin-coated plates (Figures 2(a)–2(d)).

### 3.3. Examination of Cross-Reactivity between Lectins

To examine the strength of these anti-lectin antibodies and their cross-reaction with specific and nonspecific plant lectins, we reacted each antibody with its own lectin and five other lectins. For example, we measured the reactivity of anti-WGA antibody with WGA, SBA, PNA, PHA, lentil lectin, and pea lectin. The data presented in Table 2 shows that the strongest reaction was observed between each antibody with its specific lectin, but with significant cross-reactivity with the other lectins that were not used for the preparation of antibodies. Case in point, the anti-WGA antibody reacted strongly (index 3.8) with WGA, moderately with pea lectin and PHA, but not at all with lentil lectin and SBA. On the other hand, the anti-SBA antibody reacted strongly with SBA, lentil lectin, PNA, and pea lectin with indices ranging from 3.72 to 3.79, but not with WGA (Table 2). These results indicate that there is significant cross-reactivity between different lectins.

### 3.4. Detection of Percentage of Elevation of Antibodies against Different Lectins/Agglutinins

**Figure 3:**
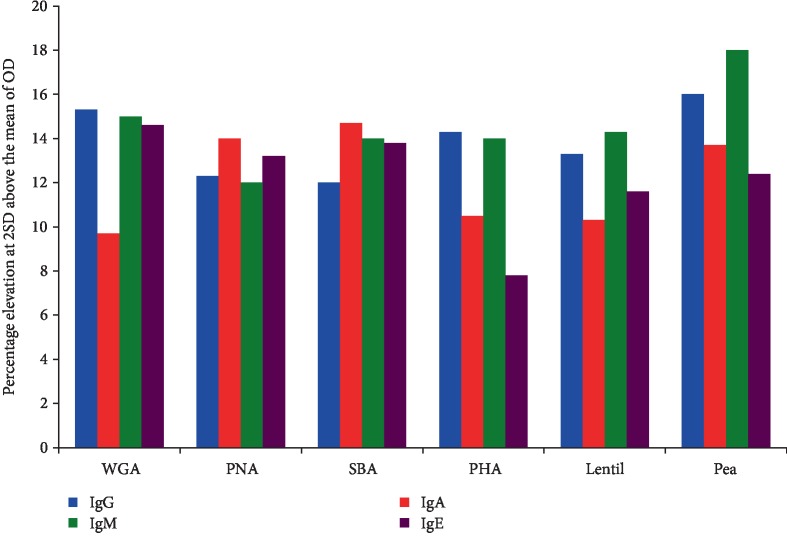
Percentage elevation of IgG, IgA, IgM, and IgE antibodies against WGA, PNA, SBA, PHA, lentil lectin, and pea lectin.

Sera from 500 healthy blood donors were screened for the presence of IgG, IgA, IgM, and IgE antibodies against WGA, PNA, SBA, PHA, lentil lectin, and pea lectin. The percentage elevation of these antibodies at 2 SD above the mean of all tested samples are presented in Figure 3.

**Figure 4:**
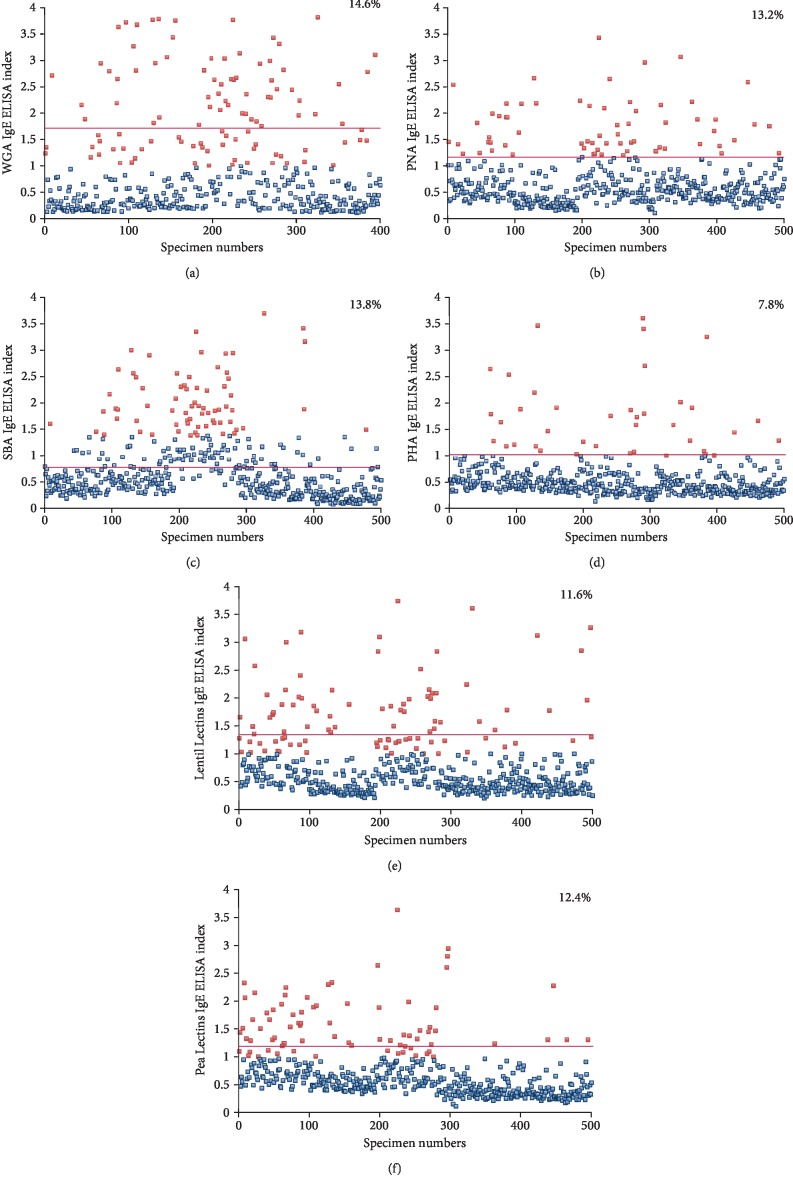
Prevalence of IgE antibodies against 6 different lectins in 500 samples. Results for IgE against (a) WGA, (b) PNA, (c) SBA, (d) PHA, (e) lentil lectin, and (f) pea lectin in the sera of 500 blood donors expressed as ELISA index with percentages of elevation. Cutoff points (red lines) are calculated from the mean + 2 standard deviation of each group.

For WGA at the cutoffs of 1.3 for IgG, 0.84 for IgA, 1.4 for IgM, and 1.68 for IgE, 15.3%, 9.7%, 15%, and 14.6% of the tested samples, respectively, had significant elevations in these antibodies. For PNA at the cutoffs of 1.46 for IgG, 1.36 for IgA, 1.45 for IgM, and 1.2 for IgE, 12.3%; 14%; 12%; and 13.2%, respectively, showed an elevation in the antibody levels. For SBA at the cutoffs of 1.25 for IgG, 1.29 for IgA, 1.45 for IgM, and 1.3 for IgE, 12%, 14.7%; 14%; and 13.8%; respectively, showed an elevation in the antibody levels. For PHA at the cutoffs of 1.43 for IgG, 1.02 for IgA, 1.61 for IgM, and 1.0 for IgE, 14.3% showed elevation for IgG, 10.5% for IgA, 14% for IgM, and 7.8% for IgE isotype antibodies. For lentil lectin, at the cutoffs of 1.45 for IgG; 1.42 for IgA; 1.41 for IgM; and 1.33 for IgE, 13.3% showed an elevation for IgG, 10.3% for IgA, 14.3% for IgM, and 11.6% for IgE isotype antibodies. For pea lectin, at the cutoffs of 1.28 for IgG, 1.49 for IgA, 1.64 for IgM, and 1.23 for IgE, 16% showed an elevation for IgG, 13.7% for IgA, 18% for IgM, and 12.4% for IgE isotype antibodies. These percentages of elevation and cutoff points for each isotype were calculated from the means of the results from 500 samples plus 2 SD. A representative scattergram for only IgE with the percentage elevations for each of the 6 lectins is shown in Figure 4.

Comparing the elevations for each isotype antibody among the 6 lectins, the highest to lowest for IgG, respectively, were pea lectin, WGA, PHA, lentil lectin, PNA, and SBA. The highest to lowest for IgA, respectively, were SBA, PNA, pea lectin, PHA, lentil lectin, and WGA. The highest to lowest for IgM, respectively, were pea lectin, WGA, lentil lectin, SBA and PHA equally, and PNA. The highest to lowest for IgE, respectively, were WGA, SBA, PNA, pea lectin, lentil lectin, and PHA.

### 3.5. Comparison of IgG and IgM Antibodies against Different Lectins in Samples Negative or Positive for RF

**Figure 5:**
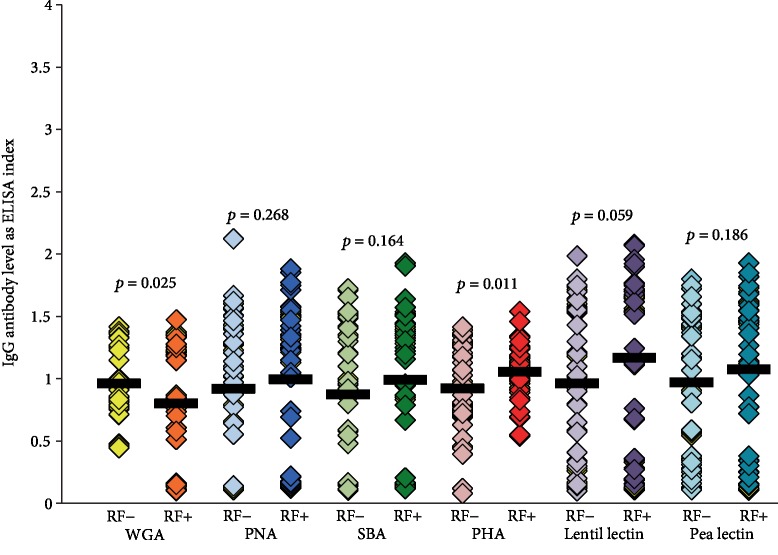
Comparison in the level of IgG antibodies against six different lectins in sera negative or positive for RF by ELISA. Aside from some elevation in IgG antibody against lentil lectin (*p* = 0.059) and PHA antibody (*p* = 0.011) in the RF-positive group, the differences in IgG antibody levels against WGA, PNA, SBA, and pea lectins when the RF-negative group was compared to the RF-positive group were insignificant, as were the correlation coefficients between these determinations.

**Figure 6:**
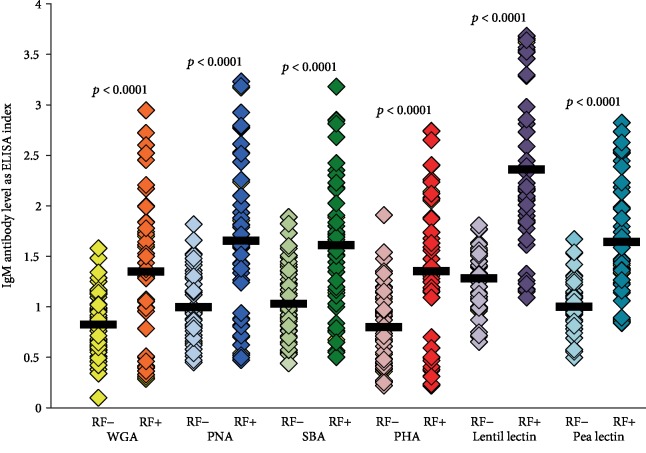
Comparison in the level of IgM antibodies against six different lectins in sera negative or positive for RF by ELISA. The IgM antibody level against all six lectins was much higher in the RF-positive group than that in the RF-negative samples (*p* < 0.0001). These results also showed significant correlations between IgM antibodies and lectins with elevated RF.

IgG and IgM antibodies against six different lectins were measured in 48 sera with normal levels and 48 sera with high levels of RF. Data presented in Figure 5 shows that other than some elevation in IgG antibody against lentil lectin (*p* = 0.059) and PHA antibody (*p* = 0.011) in the RF-positive group, nonsignificant differences in the levels of IgG antibody against WGA, PNA, SBA, and pea lectins were detected when the RF-negative group was compared to the RF-positive group. Furthermore, nonsignificant correlation coefficients between these determinations were detected (see Table 3). When a similar comparison was made between the levels of IgM antibody against these six lectins in RF-negative sera versus RF-positive sera, the IgM antibody level against all six lectins was much higher in the RF-positive group than that in the RF-negative samples (*p* < 0.0001) (see Figure 6). These results also showed significant correlations between IgM antibodies and lectins with elevated RF. These relationships between IgM antibody against lectins and abnormal RF are summarized in Table 3 (*r* = 0.46 − 0.81). This IgM correlation in RF-positive samples was the most significant with lentil lectin IgM (*r* = 0.81), followed by pea lectin (*r* = 0.66), SBA (*r* = 0.62), PNA (*r* = 0.56), WGA (*r* = 0.48), and PHA (*r* = 0.46). When we tested for simultaneous elevation of lectin antibodies, we found that 22 out of 48 or 46% of samples with elevated RF also exhibited an elevation of IgM antibody against all six lectins used in the study. The other specimens either did not react or reacted to some lectins but not to others.

### 3.6. Comparison of IgG Antibody against Different Lectins in Samples Negative or Positive for ANA

**Figure 7:**
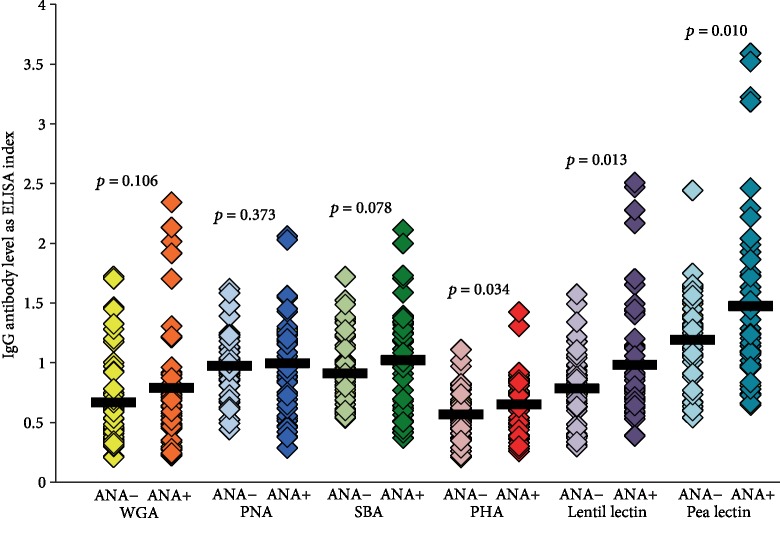
Comparison in the level of IgG antibodies against six different lectins in sera negative or positive for ANA by ELISA. IgG antibody against pea lectin, lentil lectin, and PHA, in, respectively, descending degrees, were significantly elevated in the ANA-positive group. Statistically nonsignificant differences in the levels of IgG antibody against WGA, PNA, SBA, and pea lectins were detected when the ANA-negative group was compared to the ANA-positive group.

IgG antibody against these lectins was measured in 48 samples with an ANA titer of 1 : 20 or less and in 48 additional sera with an ANA titer ranging from 1 : 80 to 1 : 1280. Data presented in Figure 6 shows that the IgG antibody against pea lectin, lentil lectin and PHA, in, respectively, descending degrees, were significantly elevated in the ANA-positive group. Statistically nonsignificant differences in the levels of IgG antibody against WGA, PNA, SBA, and pea lectins were detected when the ANA-negative group was compared to the ANA-positive group (see Figure 7). Small to moderate correlations were noted for pea lectin (*r* = 0.33) and lentil lectin (*r* = 0.20), while the other lectins showed nonsignificant correlations (*r* = 0.1 or less).

## 4. Discussion

Antibodies to dietary antigens including lectins and agglutinins can be found in the sera of some healthy subjects, indicating that intact lectins entered the blood after the host consumed vegetables, fruits, or nuts containing lectins [12, 22, 41–44]. This is because lectins have a globular tertiary structure that makes them resistant to degradation by digestive enzymes or heat, as well as a lack or unavailability of cleavage sites for proteases of the digestive tract [45]. These lectins in the blood can mediate a variety of biological effects, such as immune response and the production of lectin-specific antibodies [13, 22, 26, 27, 42, 43]. However, when we did a Med search for immune reaction or antibodies against lectins/agglutinins, we found only our own earlier investigation about PNA antibody [22] plus five additional articles published between 1986 and 1998 about measuring IgG or IgA antibodies against dietary lectins in human blood [26, 27, 42, 46, 47].

Therefore, the major goal of this study was to measure the degree of immune reactivity of antibodies made against different lectins with 62 different tissue antigens and peptides. The idea for this arose from our previous study [48] in which we found a correlation between the WGA antibody and the presence in human blood of antibodies against a variety of tissues, particularly the brain, adrenal gland, heart, joint, ovary, and pancreas. For example, when WGA-positive sera were tested for the presence of tissue antibodies and compared to WGA-negative sera, 68% of WGA-positive sera reacted to the same antigens, while only 16% of WGA-negative individuals reacted to brain antigens. This led us to believe that WGA-specific antibodies in the sera of some patients were cross-reacting with human tissue antigens [49]. To examine this possible cross-reactivity between different lectins and human tissue, we purchased affinity-purified lectin-specific antibodies and examined their direct reactivity with a variety of human tissue antigens (see Table 1). As could be expected, anti-WGA antibodies bound to WGA with a very strong index of 3.8. In comparison, anti-WGA antibodies reacted with 34 out of 62 tested antigens with indices ranging from 0.43 (borderline), with *α*+*β* tubulin, to as high as 3.4 (very strong) with TPO. Likewise, anti-PHA antibodies reacted with 20 out of 62 tested antigens, with TPO also being the highest, with an index of 3.6 (very strong). It is important to note that unimmunized goat serum did not react with any of the 62 tissue antigens used in this study. Furthermore, in comparison to anti-PNA or anti-SBA antibodies binding to PNA or SBA resulting in indices of about 3.8, the reaction of anti-PNA and anti-SBA antibodies binding to human tissue resulted with no binding with the majority of the tissue antigens and low or borderline binding with the others, with the exception of anti-SBA antibodies binding to tyrosinase with an index of 1.6 (moderate). This indicates that both PNA and SBA share a minor cross-reactivity with some human tissue antigens. However, strong reactions of anti-WGA, anti-PHA antibodies, or both with TPO; strong reactions of anti-WGA antibodies with zonulin, insulin islet cells, ovary, and calmodulin; and moderate reactions of anti-WGA antibodies with *α*-myosin and several other tissue antigens indicate strong cross-reactivity between WGA and a variety of tissue antigens (Table 1). Earlier studies showed that the peripheral nerve and thyroid nodules had strong affinity to both WGA and concanavalin-A [49, 50]. This binding of lectins to different cells' surface glycoprotein may be responsible for lectin-induced autoimmunity (LAM) [47]. As early as 1988, Kitano et al. [51] made an attempt to detect islet cell surface antibodies. WGA-bound islet-cell glycoproteins were incubated with the sera of patients with insulin-dependent diabetes mellitus; strong IgG immunoreactivity was found in 38% of patients and 7% of controls. A very strong reaction between WGA- and PHA-specific antibodies with recombinant TPO or other antigens may indicate that molecular mimicry between lectins and tissue antigens is a possibility. Additionally, anti-WGA reaction with PHA and anti-PHA reaction with WGA (shown in Table 2) further support cross-reactivity between different lectins. Further confirmation of this cross-reactivity in future studies would mean serious implications about the direct role that lectins play in autoimmune diseases.

Although thyroid autoantibodies are already used for confirming the diagnosis of thyroid autoimmunities [52–54], many infectious agents that share homologies with the thyroid tissue, such as *Yersinia enterocolitica*; *Helicobacter pylori*; *Candida albicans*; *Borrelia burgdorferi*; and, as our own study now demonstrates, the lectins WGA and PHA, may contribute to the presence of these antibodies in the blood [55–57]. In relation to homologies between lectins and the human tissue, Toda et al. in 1985 compared the amino acid sequences of brain calmodulin with wheat germ, scallop, and spinach calmodulin and found about 90% similarities between mammalian and plant calmodulins [58]. This amino acid homology between the brain and WGA calmodulin may explain the strong reaction found in our study between anti-WGA and calmodulin and the moderate reaction found between the anti-PHA antibody and calmodulin (Table 1).

In addition to calmodulin, WGA also showed structural similarities (25-35% homology) with other tissue antigens such as fibulin, fibrillin, and tenascin from human placenta. These tissue antigens have also been shown to have immunochemical reactions with anti-PHA, anti-WGA, and anti-ConA antibodies [59–61].

Generally, AA sequence homology, domains sharing similar topology, and protein misfolding are thought to be responsible for the production of cross-reactive antibodies [62, 63]. We found that the anti-WGA antibody had a strong reaction with the ovary, a moderate reaction with heart *α*-myosin, and a low reactivity with platelet glycoprotein and fibulin, which is also expressed in the heart. The anti-PHA antibody had a moderate reaction with *α*-myosin and low reactions with platelet glycoprotein and fibulin. Anti-SBA antibody had a low reaction with *α*-myosin, and borderline reactions with ovary and platelet glycoprotein. The anti-PNA antibody had borderline reactions with fibulin, the ovary, and platelet glycoprotein. Therefore, our findings confirm the immunoreactivity of anti-lectin antibodies in varying degrees with fibulin, the ovary, and *α*-myosin. This could be related to the expression of common antigens in tissues such as the placenta, ovary, or heart [59, 60].

We also found two or more lectins that reacted with several tissue antigens simultaneously, for example, the reaction of anti-WGA and anti-PHA antibodies with TPO and calmodulin (Table 1). This simultaneous reaction of different lectin antibodies with the same tissue antigen could be related to a high degree of homology in the putative carbohydrate recognition domains [64, 65].

Our findings regarding the immunoreactivities of two out of four different lectin antibodies with many tissue antigens focus our future analytical efforts towards presenting further evidence for the postulated homologies between different lectins and human tissue antigens. The second goal of our study was to measure the prevalence of IgG, IgA, IgM, and IgE antibodies against six different lectins isolated from wheat (WGA), peanut (PNA), soy (SBA), red kidney bean (PHA), lentil, and pea in 500 healthy volunteers. At 2 SD above the mean, we detected high levels of antibodies in 10-16% of the blood donors. This indicates that in a significant percentage of the population, lectins, glycoproteins, or peptides enter into the circulation due to inability to digest lectins and agglutinins and failure of oral tolerance. Immune response against these proteins or peptides may result in IgE isotype antibody in some and IgG, IgA, or IgM isotype antibody in others. Production of IgE antibody against various lectins detected in 8-14% of blood donors may indicate classical type-1 allergic reaction to these lectins (Figure 4). While an elevation of IgG, IgA, or IgM antibodies against these lectins may not indicate allergy, in the context of molecular mimicry or cross-reactivity, these antibodies may contribute to autoimmune reactivities.

In order to examine the relationship between lectins and autoimmunities, our third goal was to measure IgG, IgM, or both against different lectins in sera with low or very high levels of RF or ANA, which are considered biomarkers of rheumatoid arthritis and other autoimmune disorders. Interestingly, our data summarized in Figures 5–7 and Table 3 showed strong correlations between the levels of IgM but not IgG against six different tested lectins in RF-positive sera, but not in RF-negative samples. Although the RF level in our study ranged from 101 to 342 IU/mL, we did not observe a one-to-one relationship between IgM lectin antibody and IgM anti-aggregated IgG or level of RF. However, as mentioned in the introduction, IgG from the sera of RA patients were found to have 50-fold the binding capacity to lectins as IgG from controls [25]. Although we found high levels of IgM anti-lectin antibody in sera with elevated RF, this does not tell us if exposure to lectins is responsible for the induction of RF formation in humans.

Similar measurements of the IgG antibody against six different lectins in ANA-negative and ANA-positive sera showed no correlation or very low correlation (*r* = 0.020, 0.33) with only two out of the four lectins (Figure 6). This elevation in the IgG antibody against lentil lectin and pea lectin in about 20% of the ANA-positive samples was independent of the RF level, because when we compared the RF levels in these samples, only one out of ten sera showed elevated RF. However, overall correlation between RF and lectin IgM antibody, but not lectin IgG antibody, with both elevated RF and ANA may indirectly indicate that lectins play a role in the production of RF, or the aggregation of IgG, and the formation of IgM anti-aggregated IgG. Additionally, the detection of high IgM antibody against all six different lectins in 22 out of 48 specimens with elevated RF may indicate that lectins play a significant role in the production of RF and, possibly, rheumatoid arthritis in a subgroup of subjects.

Finally, the presence of lectin antibodies in about 15% of blood donors reinforces the hypothesis that undigested lectins can penetrate the gut barriers, thus stimulating an immune response against the specific anti-lectin antibodies and the many tissue antigens shown in this study. The successful contact of these antibodies with their target tissues may initiate the process of autoimmune reactivity. If future research further confirms the contribution of lectins to autoimmunities, then the presence of these lectin antibodies in the blood may serve as a guide for the removal of lectins/agglutinins from a patient's diet, preventing lectin-associated autoimmune diseases.

These findings are greatly different from the unjustified claims in a book that was published in 2017. In *The Plant Paradox* [66], Dr. Steven R. Gundry argues that lectins—a group of naturally-occurring proteins found in nearly all plants–are the root cause of most illnesses plaguing modern society and therefore should be completely removed from our diet. We strongly disagree with this kind of misleading blanket condemnation of all lectins at all times everywhere. Based on our results, we do posit that in individuals with failure of oral tolerance to lectins and agglutinins, the passage of these undigested plant antigens and peptides into the blood may indeed cause a myriad of serious problems, including autoimmune disorders. Thus, the production of high levels of antibodies against these molecules may serve as a guide for the management of patients with lectin-related autoimmunities. Clinical trials would be needed to determine correlations between given anti-lectin antibodies and particular autoimmune diseases.

## 5. Conclusions

In summary, our study shows that lectin-specific antibodies react with a variety of human tissue antigens. The fact that lectin IgG, IgM, and IgE antibodies were detected in 8-15% of our blood samples supports the assertion that undigested lectins and agglutinins can cross the gut barriers, after which, they can bind to IgG, resulting in its aggregation and formation of levels of IgM antibodies but not IgG against different lectins in sera with elevated levels of RF. Our results thus indicate that lectins or the antibodies produced against them may contribute directly or indirectly to autoimmunity. More information about lectins and other foods and their roles in autoimmunity can be found in “Food Immune Reaction And Autoimmunity,” a special issue of Alternative Therapies in Health and Medicine [67].

Of course, our experiments have their limitations, as all experiments do. Although we did perform serial dilution studies for specificity and inhibition studies with results shown in Figures 1 and 2, we did not do experiments to find out whether the addition of anti-lectin antibodies to the plates coated with tissue antigen such as TPO in the presence or absence of sugars such as N-acetylglucosamine, methyl-mannoside, D-galactose, and others inhibits the reaction. Additional experiments can always be done to improve testing conditions, and it is hoped that this current work will inspire future studies that will build upon what we have begun here.

## Figures and Tables

**Table 1:** Reaction of unimmunized goat serum and affinity-purified polyclonal goat anti-lectin antibodies with different tissue antigens expressed as ELISA index.

Antigen	Controls	WGA	PNA	SBA	PHA
Parietal cell	0.41 -	1.00 ++	0.42 -	0.41 -	0.29 -
Intrinsic factor	0.26 -	1.10 ++	0.37 -	0.40 -	2.00 +++
ASCA+ANCA	0.34 -	1.80 +++	0.43 +	0.55 +	0.40 -
Calprotectin	0.17 -	1.00 ++	0.46 +	0.29 -	1.80 +++
DPP-IV	0.38 -	1.10 ++	0.22 -	0.41 -	0.81 ++
21-Hydroxylase	0.22 -	0.85 ++	0.41 -	0.33 -	0.22 -
Hepatocytes	0.33 -	1.30 ++	0.39 -	0.56 +	2.30 ++++
Somatotropin	0.48 +	1.90 +++	0.50 +	0.60 +	0.90 ++
Enteric nerve	0.21 -	0.42 -	0.72 +	0.50 +	0.22 -
Zonulin	0.39 -	2.70 ++++	0.37 -	0.60 +	0.31 -
MBP	0.40 -	1.75 +++	0.60 +	0.64 +	0.71 +
*α*+*β* Tubulin	0.17 -	0.50 +	0.32 -	0.21 -	0.72 +
Neurofilaments	0.35 -	1.10 ++	0.18 -	0.37 -	1.42 +++
Asialoganglioside	0.31 -	0.95 ++	0.48 +	0.36 -	2.90 ++++
Synapsin	0.24 -	0.80 +	0.60 +	0.42 -	0.32 -
Human aquaporin	0.33 -	0.90 ++	0.51 +	0.38 -	0.30 -
Amyloid-*β*	0.15 -	1.10 ++	0.35 -	0.44 +	0.20 -
Neurotrophin	0.23 -	1.70 +++	0.26 -	0.60 +	0.85 ++
Neurocrescin	0.18 -	2.20 ++++	0.25 -	0.46 +	0.20 -
Rabaptin-5	0.24 -	0.84 ++	0.50 +	0.45 +	0.20 -
Presenilin	0.11 -	1.50 +++	0.80 +	0.67 +	0.53 +
Tau protein	0.16 -	1.80 +++	0.20 -	0.32 -	0.60 +
BDNF	0.18 -	0.81 ++	0.21 -	0.48 +	1.30 ++
Beta-NGF	0.25 -	1.10 ++	0.22 -	0.31 -	0.80 +
GAD-65	0.10 -	0.50 +	0.44 +	0.37 -	0.19 -
Tyrosinase	0.26 -	1.20 ++	0.28 -	1.60 +++	1.52 +++
TPO	0.39 -	3.40 +++++	0.44 +	0.90 ++	3.60 +++++
Enolase	0.41 -	2.00 +++	0.33 -	0.47 +	0.71 +
Elastin	0.20 -	0.96 ++	0.22 -	0.41 -	0.38 -
Calmodulin	0.29 -	2.45 ++++	0.31 -	0.53 +	1.34 +++
Platelet glycoprotein	0.36 -	1.30 ++	0.38 -	0.63 +	1.10 ++
Fibulin	0.34 -	1.10 ++	0.55 +	0.35 -	1.00 ++
Ovary	0.21 -	2.36 ++++	0.78 +	0.65 +	0.42 -
Insulin+islet cell	0.19 -	2.31 ++++	0.80 +	0.70 +	0.41 -
*α*-Myosin	0.37 -	2.00 +++	0.45 +	0.88 ++	1.62 +++
Acetylcholine-R	0.32 -	1.43 +++	0.50 +	0.41 -	0.42 -
Dopamine-R	0.17 -	1.90 +++	1.00 ++	0.58 +	1.10 ++
Glutamate-R	0.21 -	0.99 ++	1.10 ++	0.37 -	0.50 +
Other tissues	0.26 ± 0.18	0.21 ± 0.13	0.19 ± 0.15	0.22 ± 0.16	0.28 ± 0.17

-	+	++	+++	++++	+++++
0.00-0.42	0.43-0.80	0.81-1.3	1.31-2.0	2.1-3.0	>3
Negative	Borderline	Low	Moderate	Strong	Very strong

**Table 2:** Reaction of different anti-lectin antibodies with different lectins/agglutinins.

Agglutinins	Antibody to WGA	Antibody to PNA	Antibody to SBA	Antibody to PHA
WGA	3.8	0.55	0.44	3.56
PNA	0.72	3.8	3.79	3.1
SBA	0.3	3.4	3.76	2.63
PHA	1.7	0.95	3.0	3.64
Lentil lectin	0.46	3.7	3.78	3.71
Pea lectin	1.3	3.2	3.72	3.55

**Table 3:** Correlation between rheumatoid factor (RF) and lectin/agglutinin antibodies.

	IgM		IgG	
	Abnormal RF (101-336 IU)		Abnormal RF (101-336 IU)	
Lectins/agglutinins	Coefficient	*p* value	Coefficient	*p* value
WGA	0.48	<0.0001	0.06	NS
PNA	0.56	<0.0001	0.08	NS
SBA	0.62	<0.0001	0.06	NS
PHA	0.46	<0.0001	0.08	NS
Lentil lectin	0.81	<0.0001	0.16	0.059
Pea lectin	0.66	<0.0001	0.10	NS

## Data Availability

Data is available upon request and may be obtained by contacting the corresponding author.
